# Application of tirofiban in patients with acute myocardial infarction complicated with diabetes and undergoing emergency interventional therapy

**DOI:** 10.12669/pjms.38.1.4545

**Published:** 2022

**Authors:** Xiuying Tang, Runjun Li, Lixiang Ma, Ting Zhang

**Affiliations:** 1 Xiuying Tang, Department of Cardiology, The First Hospital of Qinhuangdao, Qinhuangdao, 066000, Hebei, China; 2 Runjun Li, Department of Critical Care Medicine, People’s Hospital of Yangjiang, Yangjiang, 529500, Guangdong, China; 3 Lixiang Ma, Department of Cardiology, The First Hospital of Qinhuangdao, Qinhuangdao, 066000, Hebei, China; 4 Ting Zhang, Geriatrics Department, Baoding First Central Hospital, Baoding, 071000, Hebei, China

**Keywords:** Acute ST-segment elevation myocardial infarction, Diabetes, Percutaneous coronary intervention, Cardiac function

## Abstract

**Objectives:**

To investigate the application of tirofiban in patients with acute myocardial infarction complicated with diabetes and undergoing emergency interventional therapy.

**Methods:**

Two hundred patients with acute ST-segment elevation myocardial infarction (STEMI) complicated with diabetes who underwent percutaneous coronary intervention (PCI) and found to have high thrombus burden in coronary artery admitted to our hospital from September 2018 to September 2020 were selected as subjects, and were divided into two groups according to the randomization method: the intravenous tirofiban bolus group and the intracoronary tirofiban bolus group, with 100 cases in each group. The levels of LVEF, LVESD and LVEDD were detected immediately after admission and 15 days after therapy, and the enzyme-linked immunosorbent assay was utilized to detect the levels of CK-MB, MMP-9 and hs-CRP. Furthermore, the levels of BNP, TNI, CR and UREA of the patients were analyzed, and the levels of ESR and FIB were detected with an automatic blood rheology analyzer to analyze the TIMI classification and the incidence of MACE in the two groups.

**Results:**

Significant differences were seen between the two groups in the levels of various indicators after therapy. Fifteen days after therapy, the levels of LVEF and LVEDD were higher and the level of LVESD was lower in the intracoronary tirofiban bolus group than in the intravenous tirofiban bolus group (p<0.05); 3d after therapy, the levels of CK-MB, MMP-9 and BNP in the intracoronary tirofiban bolus group were lower than those in the intravenous tirofiban bolus group (p<0.05); 3d after therapy, the levels of TNI (p<0.05), CR and UREA in the intracoronary tirofiban bolus group were lower than those in the intravenous tirofiban bolus group, with no statistical difference (p>0.05); Similarly, 3d after therapy, the levels of TNI, Cr and Urea, as well as ESR, FIB and hs-CRP were lower in the intracoronary tirofiban bolus group than in the intravenous tirofiban bolus group (p<0.05). Compared with the intravenous tirofiban bolus group, the intracoronary tirofiban bolus group had a lower number of patients with Grade-0 and Grade-1, but a higher number of patients with Grade-2 and Grade-3 (p<0.05); Moreover, the incidence of MACE in the intracoronary tirofiban bolus group was lower than that in the intravenous tirofiban bolus group (p<0.05).

**Conclusion:**

In patients with STEMI complicated with diabetes who underwent PCI and found to have high thrombus burden in coronary artery, intracoronary bolus of tirofiban boasts superior therapeutic efficacy over intravenous bolus of tirofiban in significantly improving cardiac function, reducing myocardial cell damage, and improving renal function and myocardial inflammation of patients.

## INTRODUCTION

In the wake of the rapid development of today’s society, diabetes, as a frequently-occurring disease, seriously endangers public health. It has been shown in foreign studies that patients with diabetes are more prone to acute myocardial infarction (AMI), with a prevalence of 3 to 5 times that of non-diabetic patients.^1^ Patients with diabetes mellitus complicated with type myocardial infarction usually suffer from a higher mortality rate. In addition, abnormal glucose metabolism is also one of the primary reasons for atherosclerosis.^2^ Diabetes has an inextricable connection with cardiovascular diseases risk equivalents, in which about 80% of patients with diabetes die of cardiovascular diseases.^3^ Emergency percutaneous coronary intervention (PCI) has been applied as the principal method for the treatment of AMI, but it is often accompanied by myocardial hypoperfusion triggered by postoperative slow bleeding and no reflow.^4^ Emergency PCI, as a proven therapy in various clinical trials, is characterized by multiple effects such as effective unblocking infarction of the associated vessels, recovery of myocardial reperfusion, effective rescue of ischemic myocardium, and improvement of long-term prognosis. A majority of patients with diabetes complicated with myocardial infarction have severe coronary artery lesions, no intraoperative reflow, insufficient myocardial perfusion, and increased probability of stent thrombosis postoperatively, which greatly affects the prognosis of patients.^5^ Receptor blockers are capable of significantly improving the clinical prognosis and myocardial perfusion of patients with acute STEMI in the infarct zone. However, a significant increase can be observed in the expression of platelet glycoprotein receptor numbers in patients with acute STEMI during the acute phase.^6^ From this point of view, patients with acute STEMI have more demand for platelet glycoprotein receptor blockers, among which tirofiban is a novel platelet GP II b/III a receptor antagonist with strong anti-platelet efficacy.^7^ In this paper, tirofiban was selected for the treatment of patients with acute STEMI complicated with diabetes who underwent PCI and found to have high thrombus burden in the coronary artery, and the efficacy of different bolus modalities in the application was analyzed.

## METHODS

Two hundred patients with acute STEMI complicated with diabetes who underwent PCI and found to have high thrombus burden in coronary artery admitted to our hospital from September 2018 to September 2020 were prospectively selected as subjects, and were divided into two groups according to the randomization method: the intravenous tirofiban bolus group and the intracoronary tirofiban bolus group, with 100 cases in each group. There were 67 male patients and 33 female patients in the intravenous tirofiban bolus group, aged 58-71 years, with an average age of 64.5±5.2 years. There were 69 male patients and 31 female patients in the intracoronary tirofiban bolus group, aged 56-70 years, with an average age of 63.1±5.5 years. No statistical difference can be seen in the general data of the two groups, which were comparable. All patients and their families in this study have signed the informed consent. This study was carried out under the approval of the Ethics Committee of our hospital.

### Ethical Approval:

The study was approved by the Institutional Ethics Committee of Baoding First Central Hospital (No.201601B003; Date: July 19, 2016), and written informed consent was obtained from all participants.

### Inclusion criteria:

1. Patients diagnosed by coronary angiography as meeting the indications for em
2. Patients whose ischemic symptoms worsened within 48 hour;
3. Patients with onset time less than two hour;
4. Patients with significant elevation of myocardial markers.

### Exclusion criteria:

1. Patients with severe liver and kidney dysfunction;
2. Patients who underwent PCI and coronary artery bypass grafting within half a year;
3. Patients with uncontrollable hypertension;
4. Patients with recent major operations, bleeding, and blood transfusion;
5. Patients with hematological diseases;
6. Patients allergic to tirofiban;
7. Patients with severe liver and kidney dysfunction;
8. Patients with diabetic ketosis.

Patients with diabetes, referring to the 2019 Diagnosis and Classification of Diabetes Mellitus,^9^ are defined as those with diabetic symptoms, fasting blood glucose > 7.0 mmo/L or 2 h blood glucose > 11.1 mmo/L in a glucose tolerance test, or those who have been diagnosed with diabetes and have been treated.

All patients in the two groups were given 300 mg Bayaspirin in the form of chewing and 100 μg/kg heparin intravenously prior to PCI. Other drugs, such as metoprolol, nitrates and statins, were given as usual. Patients in the intravenous tirofiban bolus group were given an intravenous bolus of tirofiban at a dose of 10 μg/kg (purchased from Shandong New Time Pharmaceutical Co., Ltd., State Drug Approval No.: H20090227, Strength: 12.5 mg) prior to PCI. In contrast, patients in the intracoronary tirofiban bolus group received a bolus of 10 μg/kg of tirofiban via the coronary artery prior to PCI. In both groups, tirofiban was continuously pumped into the vein with the loading dose of 0.15 μg/ (kg.min).

### Detection of the levels of LVEF, LVESD, LVEDD:

Immediately after admission, three daysafter therapy, and 15 days after therapy, 5ml of venous blood was drawn under fasting state, and the supernatant was collected by centrifugation in both groups. The Vivid E9 ultrasonography was used to evaluate the changes in cardiac function (mainly including left ventricular ejection fraction (LVEF), left ventricular end-diastolic diameter (LVEDD) and left ventricular end-systolic diameter (LVESD)) by measuring the cardiac morphology and structure of the patients immediately after admission and seven days after therapy by the same physician.

### Detection of the levels of CK-MB, MMP-9 and hs-CRP:

Enzyme-linked immunosorbent assay was adopted to detect the levels of creatine kinase isoenzyme (CK-MB), matrix metalloproteinase-9 (MMP-9), and hypersensitive C-reactive protein (hs-CRP). After diluting CK-MB, MMP-9 and hs-CRP with 50mm carbonate buffer solution, antigen processing was performed, Then they were added into the reaction well of polystyrene and covered. After 24 hour, they were washed three times at 4°C on the 2nd day and then dried. 0.1 ml of diluted samples were added into each well, and positive and negative samples were added together. The control samples were placed in the environment of 42°C for 60 minutes. After the liquid was removed, they were washed again for three times and then dried. 0.1 ml of MMP-9 and hs-CRP antibody were added into each well and placed again for 60min. After the liquid was removed, they were washed again for three times and then dried. Subsequently, the substrate solution was put into each well, mixed well, and then 0.1 ml of o-phenylenediamine was added. After shading treatment for 20 minutes, 2 mol/L H2SO4 was put into 0.05 mL of each well to terminate their reaction.

### Detection of the level of BNP:

With the aid of the Triage meterpro diagnostic instrument purchased from Biosite, USA, the level of plasma brain natriuretic peptide (BNP) was detected by a highly qualified physician in our hospital using the dry-type double-antibody sandwich fluorescence immunoassay.

### Detection of the levels of TNI, Cr and Urea:

The levels of troponin I (TNI), creatinine (Cr), and urea nitrogen (Urea) were detected with a Status FirstTM analyzer, purchased from Princeton BioMeditech, USA. The lowest detected concentration of TNI was 2 ng/L, and the normal range was < 20 ng/L.

### Detection of the levels of ESR and FIB:

A full-automatic hemorheological analyzer, Succeeder SA-9000, purchased from Shaanxi Dingshi Medical Technology Co., Ltd., was used to detect and compare the levels of erythrocyte sedimentation rate (ESR) and fibrinogen (FIB) in the two groups immediately after admission and 3 days after therapy.

### TIMI grade:

Coronary artery blood flow (TIMI grade) during coronary angiography was observed. Grade-0 indicates no perfusion and no blood flow at the distal end of vascular occlusion; Grade-1 means infiltration without perfusion, partial vascular occlusion, and contrast agent cannot fill the distal vessels; Grade-2 represents partial perfusion, in which the contrast agent completely fills the distal coronary artery but at a slower rate; Grade-3 indicates complete perfusion, with the contrast agent completely filling the distal vessels.

### Incidence of MACE:

After therapy, major adverse cardiac events (MACE), including sudden cardiac death, restenosis, angina pectoris, and recurrent myocardial infarction, were evaluated in both groups and recorded and compared by the physicians in our hospital.

### Statistical Analysis:

SPSS21.0 software was sused for processing of data.. Enumeration data were represented as %, and inter-group comparison was tested by *x^2^*. Measurement data were expressed as (x ¯±s), and inter-group comparison was tested by LSD-t; p<0.05 indicates a statistically significant difference.

## RESULTS

Immediately after admission, no statistical difference was observed in the levels of LVEF, LVEDD and LVESD of patients in the two groups (p>0.05). 15d after therapy, the levels of LVEF and LVEDD were higher and the level of LVESD were lower in the intracoronary tirofiban bolus group than in the intravenous tirofiban bolus group, with statistically significant differences (p<0.05). Table-I

**Table I:** Comparison of the levels of LVEF, LVEDD and LVESD between the two groups (x̅±s).

	LVEF (%)		LVEDD (mm)		LVESD(mm)	

Group	Immediately after admission	15d after therapy	Immediately after admission	15d after therapy	Immediately after admission	15d after therapy
Intravenous tirofiban bolus group	41.43±5.23	48.32±5.13	65.06±8.34	51.73±5.61	57.39±6.25	54.86±5.61
Intracoronary tirofiban bolus group	41.39±5.21	56.16±6.25	65.08±8.36	61.24±7.22	56.85±6.23	51.79±5.17
t	0.054	9.696	0.017	10.400	0.612	4.024
p	0.957	0.001	0.986	0.001	0.541	0.001

No statistical difference immediately after admission was observed in the levels of CK-MB, MMP-9 and BNP of patients in the two groups (p>0.05). 3d after therapy, the levels of CK-MB, MMP-9 and BNP in the intracoronary tirofiban bolus group were lower than those in the intravenous tirofiban bolus group, with statistically significant differences (p<0.05).Table-II

**Table II:** Comparison of the levels of CK-MB, MMP-9 and BNP between the two groups (x̅±s).

	CK-MB (U/L)		MMP-9 (pg/mL)		BNP(pg/mL)	

Group	Immediately after admission	3d after therapy	Immediately after admission	3d after therapy	Immediately after admission	3d after therapy
Intravenous tirofiban bolus group	11.41±1.42	8.12±0.76	409.51±46.39	261.39±29.46	437.19±51.19	353.36±34.13
Intracoronary tirofiban bolus group	11.35±1.39	6.17±0.57	407.26±43.87	227.32±24.18	435.31±48.16	237.62±25.17
t	0.302	20.530	0.352	8.939	0.268	27.290
p	0.763	0.001	0.725	0.001	0.789	0.001

Table-III shows immediately after admission, no statistical difference was observed in the levels of TNI, Cr and Urea of patients in the two groups (p>0.05). Three days after therapy, the level of TNI in the intracoronary tirofiban bolus group was lower than that in the intravenous tirofiban group, with a statistical difference (p<0.05). Moreover, the levels of Cr and Urea in the intracoronary tirofiban bolus group were lower than those in the intravenous tirofiban bolus group, but with no statistical difference (p>0.05).

**Table III:** Comparison of the levels of TNI, CR and UREA between the two groups (x̅±s).

	TNI (ng/mL)		Cr (μmol/L)		Urea(mmol/L)	

Group	Immediately after admission	3d after therapy	Immediately after admission	3d after therapy	Immediately after admission	3d after therapy
Intravenous tirofiban bolus group	0.78±0.09	0.42±0.07	106.52±11.21	84.57±8.91	7.26±0.64	5.59±0.53
Intracoronary tirofiban bolus group	0.77±0.08	0.26±0.05	107.62±11.23	82.37±8.21	7.15±0.57	5.48±0.49
t	0.831	18.600	0.392	1.816	1.284	1.571
p	0.407	0.001	0.489	0.071	0.201	0.118

Similarly immediately after admission, no statistical difference was observed in the levels of ESR, FIB and hs-CRP of patients in the two groups (p>0.05). Table-IV. Three days after therapy, the levels of ESR, FIB and hs-CRP in the intracoronary tirofiban bolus group were lower than those in the intravenous tirofiban bolus group, with statistically significant differences (p>0.05).

**Table IV:** Comparison of the levels of ESR, FIB and hs-CRP between the two groups (x̅±s).

	ESR (mm/h)		FIB (mg/dl)		hs-CRP (mg/L)	

Group	Immediately after admission	3d after therapy	Immediately after admission	3d after therapy	Immediately after admission	3d after therapy
Intravenous tirofiban bolus group	24.39±2.67	16.65±1.59	375.79±41.54	334.25±32.26	6.78±0.54	4.45±0.51
Intracoronary tirofiban bolus group	24.36±2.72	12.56±1.35	371.83±42.37	296.36±28.34	6.81±0.53	2.53±0.24
t	0.079	19.610	0.667	8.824	0.397	7.439
p	0.937	0.001	0.505	0.001	0.692	0.001

TIMI blood flow grading was compared between the two groups after 7 days of therapy. Table-V. The number of patients with Grade-0 and Grade-1 was lower, and the number of patients of Grade-2 and Grade-3 was higher in the intracoronary tirofiban bolus group than those in the intravenous tirofiban bolus group, with statistically significant differences (p<0.05).

**Table V:** Comparison of TIMI blood flow grading of the two groups after 7d of therapy [n(%)].

Group	Grade-0	Grade-1	Grade-2	Grade-3
Intravenous tirofiban bolus group	28 (28.00)	34 (34.00)	12 (12.00)	26 (26.00)
Intracoronary tirofiban bolus group	9 (9.00)	18 (18.00)	26 (26.00)	47 (47.00)
X^2^	11.971	6.523	6.368	9.514
p	0.001	0.009	0.012	0.002

The incidence of MACE between the two groups of patients was compared. Table-VI, The incidence of MACE was lower in the intracoronary tirofiban bolus group than in the intravenous tirofiban bolus group, with a statistical difference (p<0.05).

**Table VI:** Comparison of the incidence of MACE between the two groups [n(%)].

Group	Sudden cardiac death	Restenosis	Angina Pectoris	Recurrent myocardial infarction	Total incidence (%)
Intravenous tirofiban bolus group	4	8	5	7	24 (24.00)
Intracoronary tirofiban bolus group	1	3	3	4	10 (10.00)
X^2^					6.945
p					0.008

## DISCUSSION

Myocardial infarction is a severe life-threatening disease, while diabetes is a common metabolic syndrome of aging. As the population ages, a yearly increase is seen in the incidence and mortality of diabetes-related acute myocardial infarction (AMI).^10^ Emergency PCI, features its ability to open the arteries related to the infarction in a timely and effective manner and save the dying myocardium, has become the most effective mean for the treatment of acute STEMI and a crucial therapeutic measure for the rescue of acute STEMI. In case of diabetes mellitus complicated with acute myocardial infarction, a high incidence of non-reflow and slow bleeding will follow, leading to increased postoperative adverse cardiovascular events, more complications, poor prognosis and high mortality.^11^ Receptor blockers are touted to significantly improve myocardial perfusion and clinical prognosis in the infarcted area in patients with acute cross-sectional myocardial infarction. It has been shown in related studies that in patients with acute coronary syndrome, percutaneous coronary intervention can effectively reduce the occurrence of non-fatal myocardial infarction and targeted coronary angiogenesis.^12^ Tirofiban has been shown to have unique efficacy in reducing the incidence of secondary non-fatal myocardial infarction and coronary target vessel remodeling in patients with acute coronary syndrome. Relevant studies have shown that with high-dose tirofiban, a higher platelet inhibition rate can be achieved, and the clinical prognosis of patients can eventually be ameliorated.^13^

Disorder of microcirculation in patients with acute myocardial infarction can be attributed to a variety of factors, such as distal embolism, ischemia-reperfusion injury, and individual responses to microcirculation reperfusion.^14^ Coronary atherosclerosis, as the pathological basis of myocardial infarction, is often accompanied by hemorrhage, plaque rupture, thrombosis and other manifestations during its progression, leading to acute coronary occlusion and myocardial ischemic necrosis. It is a relatively common coronary heart disease in acute coronary syndrome.^15^ LVESD and LVEDD are indicators to evaluate left ventricular systolic and diastolic functions, respectively. LVEF can reflect the pumping function of the left ventricle. As shown in the results of this paper, compared with patients with intravenous bolus of tirofiban, those who received intracoronary bolus of tirofiban have higher levels of LVEF and LVEDD, and a more significant decrease in the level of LVESD, indicating that intracoronary bolus of tirofiban can significantly improve the cardiac function of patients with STEMI complicated with diabetes who underwent PCI and found to have high thrombus burden in coronary artery.

MMPs, which fall into a class of biological enzymes capable of degrading extracellular matrix (ECM), feature the function of degrading the matrix protein products in the ECM of the myocardium, further stimulating cells, and enhancing collagen synthesis, thereby leading to myocardial thickening and ventricular remodeling. MMP-9 belongs to the MMP gelatinase family and can degrade a variety of collagen and fibrin.^16^ BNP, a peptide found in the spinal cord, lungs, brain and other tissues, is discovered in higher concentrations in the heart. According to related studies, BNP will be released from the blood in the event of myocardial damage in the body. Therefore, BNP has a close bearing on myocardial infarction.^17^ As shown in the results of this study, compared with patients with intravenous bolus of tirofiban, those who received intracoronary bolus of tirofiban have significantly reduced levels of CK-MB, MMP-9 and BNP, indicating that intracoronary bolus of tirofiban may play a role in alleviating myocardial injury of patients with STEMI complicated with diabetes who underwent PCI and found to have high thrombus burden in coronary artery.

TNI is mainly found in cardiomyocytes and is consolidated on myofibrils in the form of binding proteins, a small part of which is free in the cytoplasm. Once various cardiomyocytes are damaged, TNI can quickly enter the peripheral blood circulation and improve the level of TNI in serum.^18^ Urea, as the main end product of human metabolism, can be reabsorbed in the renal tubules of each segment after being filtered out by the glomerulus. The sources of Cr in serum include endogenous pathways and exogenous pathways. The remaining Cr that is not reabsorbed by the renal tubules usually enters the primary urine. Therefore, the levels of CR and UREA in serum are recognized as the main observation indexes of renal function.^19^ As shown in the results of this study, compared with patients with intravenous bolus of tirofiban, those who received intracoronary bolus of tirofiban have significantly reduced levels of TNI, Cr and Urea, indicating that intracoronary bolus of tirofiban may play a role in alleviating the damage of myocardial cells and improving renal function of patients with STEMI complicated with diabetes who underwent PCI and found to have high thrombus burden in coronary artery.

In case of AMI symptoms, the regional myocardium at this time will show ischemia and hypoxia, resulting in regional metabolic acidosis, red blood cell aggregation in the blood, and accelerated ESR production. Consequently, ESR changes can be invoked as a criterion to judge the severity of myocardial inflammation and ischemia.^20^ Fibrinogen, an acute reactive protein secreted by liver synthesis, boasts the highest concentration of coagulation proteins. Fibrinogen can be converted to fibrin under the action of thrombin, accelerating the formation of thrombus. In view of the positive linear correlation between the amount of hs-CRP in human serum and the inflammatory response of the body and the degree of damage of related tissues, hs-CRP may become one of the principal markers for predicting cardiovascular diseases in the future.^21^ As shown in the results of this study, compared with patients with intravenous bolus of tirofiban, those who received intracoronary bolus of tirofiban have significantly reduced levels of ESR, FIB and hs-CRP, indicating that intracoronary bolus of tirofiban may play a role in inhibiting the formation of thrombosis and improving myocardial inflammation of patients with STEMI complicated with diabetes who underwent PCI and found to have high thrombus burden in coronary artery.

### Limitations of the study:

The number of subjects included in this study is limited. In addition, we only analyzed the cases included in our hospital, which may not be representative enough. We look forward to a multi-center study in the future to reach more comprehensive conclusions.

## CONCLUSIONS

In terms of the treatment of patients with STEMI complicated with diabetes who underwent PCI and found to have high thrombus burden in coronary artery, intracoronary bolus of tirofiban has advantages over intravenous bolus of tirofiban in significantly ameliorating cardiac function, alleviating myocardial cell damage, and improving renal function as well as myocardial inflammation.

### Authors’ Contributions:

**XT & TZ:** Designed this study and prepared this manuscript,and are responsible and accountable for the accuracy or integrity of the work.

**RL:** Collected and analyzed clinical data.

**LM:** Significantly revised this manuscript.
